# Using AI to Train Future Clinicians in Depression Assessment: Feasibility Study

**DOI:** 10.2196/87102

**Published:** 2026-02-12

**Authors:** Friederike Holderried, Alessandra Sonanini, Annika Philipps, Christian Stegemann-Philipps, Lea Herschbach, Teresa Festl-Wietek, Stephan Zipfel, Rebecca Erschens, Anne Herrmann-Werner

**Affiliations:** 1 TIME - Tübingen Institute for Medical Education University of Tübingen Tübingen, Baden-Wurttemberg Germany; 2 Department of Psychosomatic Medicine and Psychotherapy University Hospital Tübingen University of Tübingen Tübingen Germany; 3 German Center for Mental Health (DZPG), Partner Site Tübingen Tübingen Germany

**Keywords:** depression diagnosis, generative pretrained transformer, GPT-powered chatbot, large language model, LLM, medical students, suicidality

## Abstract

**Background:**

Depression is a major global health care challenge, causing significant individual distress but also contributing to a substantial global burden. Timely and accurate diagnosis is crucial. To help future clinicians develop these essential skills, we trained a generative pretrained transformer (GPT)–powered chatbot to simulate patients with varying degrees of depression and suicidality.

**Objective:**

This study aims to evaluate the applicability and transferability of our GPT-4-powered chatbot for psychosomatic cases. Specifically, we aim to investigate how accurately the chatbot can simulate patients exhibiting various stages of depression and phases of suicidal ideation, while adhering to a predefined role script and maintaining a sufficient level of authenticity. Additionally, we want to analyze to what level the chatbot is suitable for practicing correctly diagnosing depressive disorders in patients, as well as assessing suicidality stages.

**Methods:**

We developed 3 virtual patient role scripts depicting complex, realistic cases of depression and varying degrees of suicidality collaboratively with field experts and aligned with mental health assessment guidelines. These cases were integrated into a GPT-4–powered chatbot for practicing clinical history-taking. A total of 148 medical students, with an average age of 22.71 years and mostly in their sixth semester, interacted individually with one of the randomly assigned virtual patients through chat. Following this, they completed a questionnaire assessing their demographics and user experience. Chats were analyzed descriptively to assess diagnostic accuracy and suicidality assessments, as well as the role script adherence and authenticity of the artificial intelligence (AI). This was done to gain further insight into the chatbot’s behavior and the students’ diagnostic accuracy.

**Results:**

In over 90% (725/778) of the answers, the chatbot maintained its assigned role. On average, students correctly identified the severity of depression in 60% (81/135) and the phase of suicidality in 67% (91/135) of the cases. Notably, the majority either failed to address or insufficiently explored the topic of suicidality despite explicit instructions beforehand.

**Conclusions:**

This study demonstrates that a GPT-powered chatbot can simulate patients with depression fairly accurately. More than two-thirds of participants perceived the AI-simulated patients with depression as authentic, and nearly 80% (106/135) indicated they would like to use the application for further practice, highlighting its potential as a training tool. While a small proportion of students expressed reservations, and the overall diagnostic accuracy varied depending on the severity of the case, the findings overall support the feasibility and educational value of AI-based role-playing in clinical training. AI-supported virtual patients provide a highly flexible, standardized, and readily available training tool, independent of real-life constraints.

## Introduction

Depression affects an estimated 280 million people globally, representing one of the world’s most prevalent mental health conditions [1,2]. This disorder, along with anxiety, contributes to a US $1 trillion annual economic burden [3]. Despite the availability of structured diagnostic criteria through the *ICD-10* (*International Statistical Classification of Diseases, Tenth Revision*) and the *DSM-5* (*Diagnostic and Statistical Manual of Mental Disorders* [Fifth Edition]) [4,5], accurately identifying depression remains challenging. Symptom interpretation is often complex, as presentations can vary significantly between individuals [6]. Additionally, factors such as stigma and limited access to care contribute to the high rate of undiagnosed cases [7,8]. Effective diagnosis, therefore, requires not only familiarity with the formal criteria but also clinical expertise to contextualize symptoms within the broader patient history and presentation [6]. Worldwide, organizations have respective guidelines on diagnosing and treating depression [9-11]. Thorough patient assessment is essential for classification and therapy of depression, but also suicidality—as currently over 700,000 suicide deaths occur annually [12]. However, studies show that diagnostic accuracy is inconsistent among physicians [13-16]. Respective training is crucial to close this gap and has proven to be successful [17-22].

Artificial intelligence (AI)–simulated patients allow learners to practice clinical interviews flexibly, repeatedly, and at scale, providing realistic interactions to train diagnostic accuracy [23-25]. Our research group has already demonstrated the effectiveness of a generative pretrained transformer (GPT)–powered chatbot used as an AI-driven virtual patient for clinical history-taking exercises of somatic diseases, with the capability to provide automated feedback [26,27]. Despite these advances, empirical research on AI-driven virtual patients in the field of mental health training is still limited [28]. In particular, little is known about the stability of large language model (LLM)–generated symptom presentations, their ability to represent nuanced depressive or suicidal symptoms, or their impact on students’ diagnostic reasoning. Therefore, we developed a training module for medical students within their regular teaching in psychosomatic medicine and psychotherapy (PSM), using AI-driven virtual patients with depressive symptoms and varying degrees of suicidality. Virtual patients are well-established in medical education and have been shown to help students apply communication skills in clinical practice [29-31]. Recent advances in natural language processing have enhanced interactions with virtual patients to increase user engagement [32]. LLMs are particularly useful in mental health training and have been shown to accurately answer questions related to depression, even outperforming general practitioners in direct comparisons of treatment recommendations [17,33]. They have also been used as interviewee models for depression screening [34].

In this study, we aim to evaluate the applicability and transferability of our GPT-4–powered chatbot for psychosomatic cases, specifically addressing the following research questions: (1) How accurately can a GPT-4–powered chatbot simulate patients exhibiting various stages of depression and phases of suicidal ideation, while adhering to a predefined role script and maintaining a sufficient level of authenticity? What specific challenges and limitations can be observed? (2) To what level is the GPT-4–powered chatbot suitable for practicing correctly diagnosing depressive disorders in patients, as well as assessing suicidality stages?

## Methods

### Creation of AI-Simulated Patients

**Figure 1:**
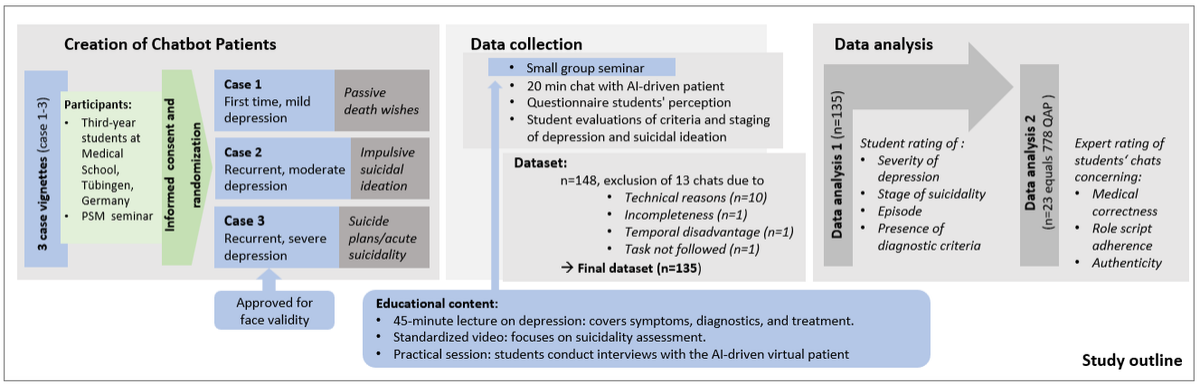
Study outline. AI: artificial intelligence; PSM: psychosomatic medicine and psychotherapy; QAP: question-answer pair.

For this study, we created 3 different virtual patient role scripts, each describing a complex, realistic patient history and current symptomatology of depression or suicidality stage (Figure 1 provides further details). The case vignettes and role scripts were collaboratively developed and critically revised by field experts and aligned with guidelines for mental health assessment in virtual patients [35]. All case vignettes were created exclusively by clinical experts and were not generated or assisted by any AI system. These patient cases were integrated into our GPT-4–powered chatbot, which was prompted for the purpose of practicing clinical history taking [26] and adapted to the new challenges within the mental health context. ChatGPT (OpenAI) was selected due to its wide availability, established performance in health-related natural language processing tasks, and strong conversational capabilities. At the time of data collection, GPT-4 was one of the leading models and the most advanced OpenAI Model. We had prior experience with GPT-4 in complex role-playing settings and were thus able to ensure a high level of quality of responses. In addition, the OpenAI account we used to access the application programming interface had a sufficiently high token limit to allow usage in groups of 10-20 people simultaneously. Temperature for GPT-4 was set to 0.1 to minimize risk of diversion from the provided information. While slightly repetitive, the answers were usually regarded as natural enough, even with this low setting.

Patient cases, student interactions, and questionnaires were conducted in German, and all examples cited in this document were translated into English via DeepL [36]. Textbox S3 and Figure S3 in Multimedia Appendix 1 [9,37] provide an exemplary chat history and screenshots of the application.

To categorize the levels of depression, we used the *ICD-10* diagnostic criteria from the World Health Organization’s classification system [5,38], which are also integrated into the curriculum at German universities (Table S1 in Multimedia Appendix 1). To classify suicidality, 4 stages have been defined, ranging from passive death wish (stage 1), suicidal ideation (stage 2), and suicide plans/preparations (stage 3) to suicide actions (stage 4) [37] (Table S2 in Multimedia Appendix 1).

### Experts

Case vignettes were developed by 2 experts in the field of PSM. Subsequently, 2 other experts evaluated the student chats, and 3 further experts with longstanding clinical experience in PSM and mental health reevaluated the vignettes diagnostically to check concordance. Finally, question-answer pairs (QAPs) were qualitatively coded for recurring errors by different experts in the field of PSM (Table S4 in Multimedia Appendix 1).

### Data Collection

Figure 1 provides detailed information about the setting, participants, and study design. The data collection took place between April and July 2024; the AI component of the session lasted 1 hour. Students, equipped with laptops, were introduced to a preconfigured web interface. Students were randomly assigned to interact with virtual patients exhibiting varying levels of depression severity (mild, moderate, and severe). Provided with general case details (setting, gender, and age of the virtual patient), they engaged in a 20-minute individual interaction with the AI-driven patient suffering from depression; the medical conversation was simulated through chat. After the digital interaction, students supplied demographic information and completed a questionnaire. The first section of the questionnaire focused on students’ subjective evaluation of the application, assessing the authenticity of the virtual patient and the likelihood of using the application regularly.

The second part of the questionnaire evaluated the students’ diagnostic accuracy. This included identifying the relevant *ICD-10* diagnostic criteria for depression, assessing the severity and recurrence of the episode, and determining the degree of suicidality.

The session concluded with an interactive discussion with a teacher from the department of PSM present, which involved reviewing correct answers to the questions and exploring additional insights into the diagnosis and treatment of depression aside from the encountered AI case.

### Prompt Engineering

Generally, we built on the techniques described in preceding publications [26,27]. Main aspects of the prompting are general descriptions of the patients’ situation and character, as well as a customizable, case-dependent list of medical categories. For each category, information is provided in the form of clinical information or as example answers. For this study, we included depression criteria and phases of suicidal ideation as categories in order to accurately describe the patients’ situation. We also expanded the general prompting to account for the required emotional depth of the simulated interviews. To increase authenticity, we adapted the prompts and the role scripts as described next.

We refined the virtual patient’s scripts by adding explanatory notes for ambiguous terms and structuring responses progressively, revealing details only upon user inquiry to enhance authenticity. To improve realism in sensitive discussions, we instructed the model to simulate patient reluctance and provided example dialogues. Additionally, we implemented safeguards to prevent prompt extraction attacks in example inputs that make the model repeat the hidden system prompt in the running chat, exposing it to the user. In our case, this would have included the complete character definition. These safeguards were thus also meant to ensure the model maintains its role without disclosing unintended information (Textboxes S1 and S2 in Multimedia Appendix 1 provide prompt engineering techniques and full prompting).

### Data Analysis

An overview of the data analysis process is presented in Figure 1. After completing data collection, all data were exported. Due to various reasons, 13 chats were excluded, leaving a total of 135 chats for inclusion in phase 1 of the data analysis.

#### Demographic Data

The dataset was analyzed descriptively in terms of demography and the students’ assessment of the severity of depression, the episode, the presence of the individual diagnostic criteria, and the stage of suicidality. Furthermore, questions concerning the authenticity of simulated patients and the likelihood of using the application in the future were evaluated descriptively. Frequencies and distributions were calculated for all variables mentioned above to provide an overview of students’ assessments and demographic characteristics.

#### Students´ Evaluation of Diagnosis and Suicidality

In data analysis phase 2, a total of 23 chats were selected out of the 135 chats for a more detailed exploration at the QAP level. To maximize issue detection, we selected chats in which students incorrectly assessed depression severity and suicidal ideation based on the classification established during the development of the role scripts. From this group, a random sample was drawn, stratified by the observed error rates across severity levels: 11 chats for moderate depression (highest error rate), 7 for mild (lower error rate), and 5 for severe depression (lowest error rate). In the 23 exemplary chats, 778 QAPs were recorded, with a median of 29 per conversation. To assess interrater reliability, 2 experts independently evaluated 23 randomly selected chats in terms of the extent to which the criteria had been addressed, not addressed at all, partially addressed, or fully addressed. The criteria evaluated were authenticity, episode, severity, suicidality, as well as the 10 diagnostic criteria of depression. Prior work shows that users interpret authenticity as a cue for sincerity, interpersonal alignment, and emotional credibility in chatbot interactions [39], which aligns closely with the evaluative focus of this study. More specifically, factors that influenced the authenticity of the interaction in this study included instances in which the response did not correspond with either the question or the prompt, or in which the interaction was considered to be inauthentic for other reasons, such as repetitions or the use of unconventional phrasing. Since the sources of error already occurred repeatedly in the selected chats, it can be assumed that the findings can also be transferred to the entire dataset. The QAPs served as the units of analysis. Both experts examined them independently in a Microsoft Excel 2408 sheet (Microsoft Corporation). They achieved Cohen κ=0.794 (QAP level) and κ=0.846 (chat level). Due to the high level of agreement, 1 rating was selected randomly. Statistical analysis was performed using SPSS (version 28; IBM Corp).

First, all QAPs were analyzed to assess the extent to which students considered the diagnostic criteria for depression and suicidality. Subsequently, the 23 selected chats were analyzed to identify reasons for incorrect statements, including insufficient discussion of the criteria, misinterpretation of the AI answers by the students, or AI-driven conversations that led to incorrect diagnoses.

#### Expert Validation of Case Vignettes

As we identified cases in which misleading prompts led to incorrect assessments and were based on content ambiguities in the prompt, the prompts were retrospectively reviewed independently by 3 additional experts in the field of psychosomatic medicine with regard to the diagnostic criteria for all 3 case vignettes. The experts received the same chat information as the participants to assess whether they could correctly identify the severity of depression and suicidality; the prompts were not modified or retested as part of this study.

#### Error Analysis and Prompt Associations

To examine patterns in students’ diagnostic thinking and deviations in the role of AI, we applied an inductive, theme-oriented coding approach based on the principles of data-driven analysis developed by Braun and Clarke [40]. Two expert raters independently used qualitative coding to identify recurring patterns in the QAPs, including errors in students’ diagnostic reasoning and role deviations, nonresponsive answers, and inappropriate repetitions. After discussing discrepancies to reach consensus, findings were summarized descriptively to highlight common issues and inform recommendations for improving AI-driven training interactions.

### Statistical Analysis

In phase 1, descriptive statistics (frequencies and percentages) were calculated for all variables to provide an overview of students’ assessments and demographic characteristics. In phase 2, all QAPs were aggregated and stored in a Microsoft Excel 2408 sheet. Two raters separately assessed authenticity and diagnostic criteria of each QAP. Cohen κ was calculated to determine the interrater reliability between the 2 raters.

Statistical analysis was performed using SPSS (version 28). Mean values, associated SDs, frequencies, and percentages were calculated. Figure generation was performed using Excel 2408.

### Ethical Considerations

The Ethics Committee of the Faculty of Medicine at University Hospital Tübingen approved the study (209/2024BO2). Participation was voluntary, with informed consent obtained; no compensation was offered for participation. All data were anonymized. All procedures were conducted in accordance with the Declaration of Helsinki and ethical guidelines for human participants. Informed consent for participation in this study was obtained verbally from all participants. Verbal consent was chosen over written consent because the study was conducted within an educational setting as part of the participants’ course activities. Additionally, since no sensitive or personally identifiable information was collected, verbal consent was deemed appropriate and approved by the Ethics Committee. Furthermore, participants also confirmed their agreement to data collection and processing by ticking the respective box on the computer. Participants were fully informed about the study’s purpose, procedures, and their right to withdraw at any time and without negative consequences before providing their consent.

## Results

### Demographic Data

A total of 148 chat datasets were collected, with 13 excluded (Figure 1), leaving 135 for analysis. Participants included 89 females, 45 males, and 1 diverse participant, with a mean age of 22.71 (SD 2.44; range 19-30) years. The majority (n=130) of the participants were in their sixth semester, 1 each in their fourth and seventh semesters, and 3 in their fifth semester. According to the curriculum, while most students have had some prior exposure to the topics of depression and suicidality, they have not yet studied them in a structured seminar format. Cohort sizes vary by topic and are clearly indicated to ensure accurate interpretation.

### Authenticity

Based on the dataset shown above (n=135), Table 1 shows that more than two-thirds of the students rated the AI-controlled presentation of the patient with depression as convincing, very convincing, or humanlike. A total of 78.52% (106/135) of respondents said they would use this application again (sometimes, often, or whenever possible).

To assess the perceived authenticity, AI adherence to the role script, and students’ chats in relation to the suspected diagnoses, 2 experts analyzed 23 selected chats in detail (Data Analysis section). Factors influencing authenticity, including frequent repetitions, inappropriate use of medical terminology, and unclear references to the prompt (65/778, 8.35%), were assessed. Based on these criteria, 87.5% (678/778) of QAPs were classified as authentic, while 12.85% (100/778) of QAPs were classified as inauthentic.

**Table 1:** Student´s opinion on the application.

Questions and responses			Frequency, n/N (%)
**How convincing was the AI^a^** **in the patient role?**			
	Not convincing	2/135 (1.48)	
	Acceptable	40/135 (29.63)	
	Convincing	54/135 (40)	
	Very convincing	36/135 (26.67)	
	Human like	3/135 (2.22)	
**How likely would it be for you to use the application if it was available on a regular basis?**			
	Not at all	7/135 (5.19)	
	Rarely	22/135 (16.30)	
	Sometimes	70/135 (51.85)	
	Often	29/135 (21.48)	
	Whenever possible	7/135 (5.19)	

^a^AI: artificial intelligence.

### Role Script Adherence

For content categorization, QAPs were classified as “relevant” (diagnostic criteria or suicidality) or “not relevant” (other medical topics or general conversation elements). A total of 49.36% (384/778) QAPs were considered relevant for diagnosis. For role conformity, expert raters assessed whether GPT-4’s responses (1) matched to the question asked and (2) remained consistent with the predefined role script. As shown in Table 2, GPT-4 deviated from the given role in 6.81% (53/778) of the QAPs.

Of the initial 6.81% (53/778) deviations, a total of 1.54% (12/778) stemmed from “not relevant” topics outside depression or suicidality (eg, other medical conditions) and were excluded from analysis. The remaining 5.27% (41/778) of “relevant’ QAPs were distributed across the 5 key diagnostic criteria: “loss of interest,” “loss of drive,” “pessimistic future prospects,” “changes in appetite,” and “impaired concentration” (Table 2).

**Table 2:** Role adherence of the chatbot.

GPT-4 roleplay outcome and QAP^a^ type			Frequency, n/N (%)
GPT^b^ stays in role			725/778 (93.19)
**GPT falls out of role, QAP concerning diagnostic criteria**			41/778 (5.27)
	Loss of interest	14/778 (1.80)	
	Loss of drive	5/778 (0.64)	
	Pessimistic future prospects	7/778 (0.90)	
	Changes in appetite	8/778 (1.03)	
	Impaired concentration	6/778 (0.77)	
	Other	1/778 (0.13)	
GPT falls out of role, QAP concerning other medical information			12/778 (1.54)

^a^QAP: question-answer pair.

^b^GPT: generative pretrained transformer.

### Qualitative Analysis

Identified erroneous criteria were qualitatively coded by the 2 expert raters to detect recurring patterns. The qualitative analysis of role deviations revealed 3 key issues (Table S4 in Multimedia Appendix 1 provides detailed examples). First, GPT-4 struggled with temporal consistency; time jumps (patient memories) or inconsistent timelines in patient history (“she has gradually started engaging in her previous leisure activities”) led to role-deviant behavior. This highlights the need to clearly separate current symptoms from previous history and to provide explicit timelines in the model. Second, inconsistency within the role scripts showed that overemphasis on isolated case details could lead GPT-4 to disproportionately amplify these aspects, resulting in misleading responses (“[My Concentration] has gotten a bit better since I started taking the new medication (…) But it’s still not as good as it used to be”). Third, we observed a tendency toward symptom aggravation, especially in mild cases, likely influenced by common training data patterns and negatively phrased diagnostic criteria. In 97% (33/34) of the analyzed misleading answers, there was a negative shift, resulting in a depiction of greater depression than intended in the prompt (Table S4 in Multimedia Appendix 1 summarizes the findings). These findings underscore the importance of neutral, precisely structured prompts to ensure stable and realistic virtual patient behavior.

### Students’ Diagnostic Accuracy

After the patient interviews, students provided a suspected diagnosis, including episode type, depression severity, and suicidality stage. Comparing their assessments (n=135) with the predefined case diagnoses showed a 94.07% (127/135) agreement on episode classification, with minor case-related deviations. Agreement on depression severity, particularly relevant for therapy decisions, averaged 60.00% (81/135). The distribution across the subgroups is particularly noteworthy: the case with severe depression reached an agreement of 100%, while the other cases reached only 40.82% (20/49; moderate depression) and 47.92% (23/48; mild depression) agreement. Regarding the phase of suicidality, agreement across all subgroups was 67.41% (91/135) on average, with a tendency toward better assessment of passive death wishes. To examine discrepancies in severity assessment, we reviewed the diagnostic criteria identified by students. Figure S1 in Multimedia Appendix 1 shows correctly recognized criteria (blue) and those incorrectly selected despite not being part of the role script (gray). For mild depression, students struggled to recognize “feelings of guilt” as present, while “reduced self-esteem” and “loss of drive” were mistakenly classified as present. In contrast, 100% correctly identified “sleep disorders” as absent. For severe depression, all main and secondary criteria were present, preventing false positives. The main challenge was identifying “impaired concentration” (28/38, 73.68% correct), while all other criteria were correctly assessed by at least 86.84% (33/38). Figure S1 in Multimedia Appendix 1 visualizes these findings.

### Expert Validation of Case Vignettes

**Figure 2:**
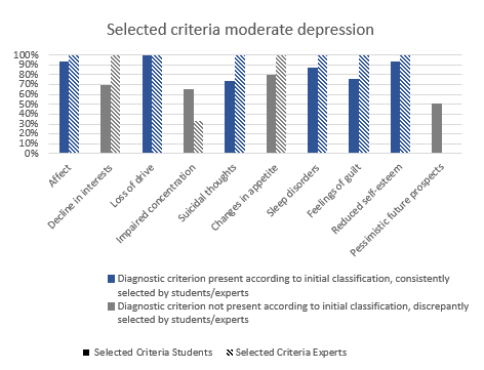
Students’ and experts’ assessment of a moderate depression case vignette. Percentages of students (monochrome bars) and experts (striped bars) who identified the displayed International Classification of Diseases, Tenth Revision (ICD-10) diagnostic criteria for depression as present in the case vignette are shown. Blue bars indicate agreement with the initial role-script classification, whereas gray bars represent criteria not originally included, highlighting discrepancies between the vignette and the standard diagnostic criteria.

Figure 2 shows the diagnostic criteria for the case developed as moderate depression. Notably, students frequently assessed “loss of interest,” “concentration disorders,” “changes in appetite,” and “pessimistic future prospects” as present, although these were not defined as present by 2 mental health field experts who had created the case vignettes. To verify this discrepancy, all 3 vignettes were reevaluated by 3 additional field experts. These experts received the same prompt as GPT-4 and assessed the virtual patient according to the diagnostic criteria. For the cases of mild depression and severe depression, there were no relevant deviations between the initial developing expert group and the reassessing expert group. For moderate depression, significant deviations occurred in individual diagnostic criteria (Figure 2). Student selections (monochrome) are contrasted with expert assessments (striped). Blue indicates agreement with the initial role script classification, while gray indicates criteria not originally included, highlighting discrepancies. Two criteria—“decline in interests” (core symptom) and “changes in appetite” (additional symptom)—were consistently rated as present by all 3 additional experts. In contrast, “impaired concentration” and “pessimistic future prospects” were selected as present by more than half of the students, while the majority of experts did not consider them to be present.

If the additional experts’ classification is used as the reference, the case originally labeled as moderate depression would instead be classified as severe. Based on this revised classification, 59% (29/49) of students correctly assessed the severity level.

### Error Analysis and Prompt Associations

Due to discrepancies in the diagnostic criteria, a detailed analysis was conducted to verify the intended diagnostic combinations for each case. Figure S2 in Multimedia Appendix 1 shows the overall and case-related results. Even with a margin of error of ±1 criterion, only for severe depression did a significant number of students (32/38) select the correct combination. For mild and revised severe (formerly moderate) depression, fewer than one-third (13/48 mild and 12/49 revised major) achieved this goal.

**Figure 3:**
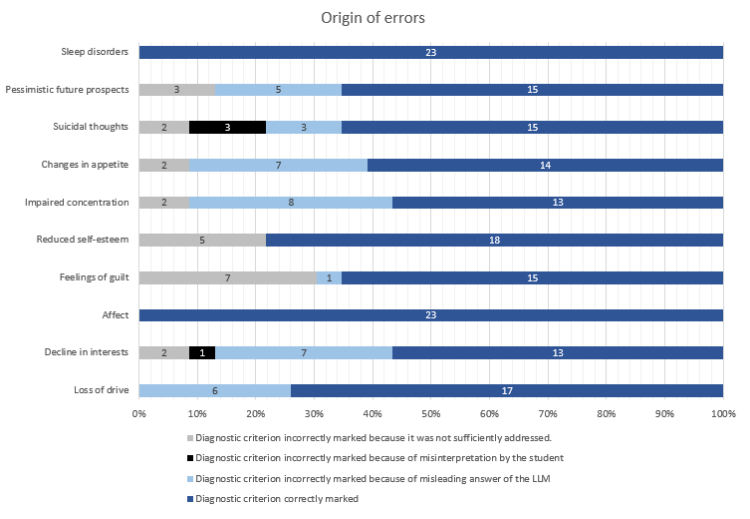
Detailed analysis of errors at the chat level. Errors were categorized according to their origin (1) errors due to insufficient addressing of the user’s query, (2) errors arising from misinterpretation of the input, and (3) errors resulting from misleading or incorrect responses generated by the large language model (LLM).

To identify error sources unrelated to case vignette creation, 2 independent experts analyzed all QAPs from 23 chats across 3 cases. Three main causes were identified (1) misleading AI responses, (2) misinterpretation of AI responses by students, and (3) insufficient exploration of the criterion by students (Figure 3).

Diagnostic criteria were assessed with varying accuracy. “Depressed mood” and “sleep disorders” were correctly identified by 100% of students, while “impaired concentration” and “decline in interests” (13/23, 56.52% each) posed the greatest challenges, mainly due to misleading AI responses. Only 1.74% (4/230) of errors resulted from misinterpretation by students, while 10.00% (23/230) resulted from insufficient follow-up questions. Correct selections also included cases in which students guessed without a solid chat-based assessment.

### Students’ Evaluation of Suicidality Stages

To analyze the assessment of suicidality in more detail, a differentiated evaluation of this part of the anamnesis interviews was carried out. This involved categorizing the information based on how the topic was addressed and an assessment by the experts regarding the degree to which the students evaluated this symptomatology. It showed that 21.74% (5/23) of the students did not address the issue of suicidality at all. Another 39.13% (9/23) did not address it sufficiently enough for a proper assessment, and only 39.13% (9/23) addressed it comprehensively enough.

## Discussion

### Overview

This study shows GPT-4’s capability to accurately simulate patients exhibiting different levels of depression and stages of suicidality in a virtual patient chat format while adhering to the role script and maintaining authenticity. Additionally, the detailed analysis of student anamnesis and their interpretation of collected patient information in relation to diagnostic criteria and suspected diagnoses reveals learning challenges inherent to the diagnostic process of depression and suicidal ideation, which this training approach helps to make more transparent and analyzable.

To ensure quality and enable further optimization, evaluating GPT-4’s role stability—especially with regard to different severity levels and stages of suicidality—is essential. Our findings suggest that the current prompting approach already provides a high degree of role stability. While in some cases inauthentic behavior was observed, these cases were rare and mostly due to structural deficiencies in the prompt design rather than inherent AI inconsistencies. Given the importance of role stability in medical applications, we propose specific recommendations for further improvements.

Our findings are consistent with previous studies [26,27] indicating that observed deviations are mainly due to structural issues in prompt design rather than from inconsistent AI responses. This suggests that case presentations can be further refined by addressing known sources of error. Other studies confirm that effective prompt engineering significantly enhances chatbot performance [41-43]. However, existing recommendations remain general, and to our knowledge, no medical education application uses a patient chatbot at this level of detail. Standard prompts like “avoid technical terms” often fail to produce consistent results. Instead, our approach relies on detailed, structured example responses, requiring careful case development but yielding significantly more precise and authentic AI-generated answers. Given the limited peer-reviewed literature, we also considered open-source online resources that document prompt engineering strategies [44].

### In-Depth Analysis of Role-Playing

Our qualitative analysis of the chats that deviated from the predefined role led to several detailed recommendations for prompt design, particularly with regard to example responses (Textbox 1). A comprehensive list is provided in Table S4 in Multimedia Appendix 1.

**In-depth analysis of role-playing**

**Temporal structure**

- Temporal changes in symptoms, especially in chronic, psychiatric, or recurrent illnesses, must be clearly structured in case vignettes. Since LLMs have difficulties with the consistent presentation of time courses, we recommend a subdivision into “current state” (eg, last 2 weeks) and “previous findings” (course of the disease), as well as the addition of a timeline to emphasize diagnostically relevant time periods.

**Formal consistency**

- Formal consistency is crucial for complex cases that require a detailed role script. Placing more emphasis on individual aspects can lead GPT-4 to overemphasize them, thereby misleading users.

**Symptom aggravation**

- We observed a tendency toward symptom aggravation, especially in mild depression. Large language models generally tend to shift toward normal variants because they reflect frequent patterns from the training data [45,46]. Possible causes are that the model associates “typical depression” with more symptoms or tends toward moderate levels, thus creating deviations from the role script. Additionally, negatively formulated diagnostic criteria (eg, “decreased energy” in *International Classification of Diseases, Tenth Revision*) may have influenced the responses. Future prompts should be neutrally formulated (“energy”) and clearly distinguish between the presence and absence of a symptom.

Overall, the AI performed convincingly, aligning with previous findings [26,27]. The suggested optimizations concern fine details, and the cost–benefit ratio depends on the application context. In virtual training, absolute accuracy is not required because real patients can also provide incorrect information due to memory errors, misunderstandings, or shame [47], and medical students need to be trained for these situations. Standardized patients, who are frequently used in medical education, also show variability, although their authenticity is often discussed as a limitation [48,49]. However, for unsupervised student feedback or exam assessments, a high degree of role stability is an essential requirement.

AI-supported virtual patients offer even greater availability, flexibility, and standardization than simulated patients [50]. A key factor for acceptance in skill learning is perceived authenticity [51,52]. In this study, over two-thirds of students rated the tool as at least “convincing,” with a further 29.62% (32/108) rating it as “acceptable,” indicating great potential for future use.

While our results confirm the high role stability of GPT-4, certain discrepancies were found in the interpretation of the severity classifications. Due to divergent assessments by our experts—particularly regarding the diagnostic criteria “loss of interest” and “appetite changes”—the original classification of the case as “moderate depression” could not be reliably maintained. To ensure clarity and accuracy, the results were reevaluated, with this case reclassified as “revised severe (formerly moderate) depression” and the results carefully contextualized in the discussion. Where reevaluation was not feasible, these data were excluded from further interpretation.

### AI Simulated Patients to Improve Students’ Diagnostic Accuracy

We found that students’ diagnostic accuracy in assessing the severity of depression depends heavily on its actual severity. While severe cases were consistently recognized, the fact that fewer than half (23/48, 47.92%) correctly diagnosed mild depression indicates deficits in the recognition of mild but clinically relevant cases. This highlights the need for more targeted training to ensure that future doctors can identify depression at all levels of severity at an early stage—especially those that would otherwise be overlooked and thus delay timely treatment.

This becomes particularly evident when examining the selection of diagnostic criteria used in forming a suspected diagnosis. Figure S2 in Multimedia Appendix 1 reveals a low success rate in correctly identifying all relevant criteria per case. Even with a margin of error of 1 criterion, only 27.08% (13/48) of students achieved this goal for mild depression and 24.49%% (12/49) for formerly moderate/revised severe depression. The results were best for severe depression: two-thirds of students selected the criteria correctly, presumably due to the higher number of symptoms present. It is important to note that students’ performance was affected by inconsistent case vignettes and AI role-playing errors, which must be taken into account when interpreting the results. In addition, they had limited time to reach a diagnosis and only little prior training on depression and suicidality. All of this should be examined more closely in future research. The difficulty of distinguishing between mild and moderate depression contributes to the lower success rates—a problem that has also been observed in general practitioners [53,54]. According to Kroenke [55], primary care physicians correctly diagnose depression only about 50% of the time, and are more likely to over-diagnose (n=15 per 100 patients) than under-diagnose (n=10) or correctly diagnose (n=10). These findings highlight that even experienced clinicians struggle with accurate classification. This underscores the need for improved diagnostic training at all levels. *ICD-10* makes this distinction even more difficult, as it often requires only a single additional minor criterion to distinguish between moderate and mild depression [38]. Mental Health cases pose an additional layer of complexity. In our experience, such cases require a higher face validity than somatic illnesses in order to compensate for potential uncertainty or overlap between individual diagnostic criteria [26,31]. A potential key improvement could be the future use of *ICD-11* (*International Classification of Diseases, 11th Revision*), which is intended to provide a less rigid discrimination of the severity levels of depression [56,57]. This was not used in this study, as it was not part of the curriculum at the time of data collection; however, it could be seamlessly integrated into the application in the future.

To further evaluate students’ diagnostic accuracy, we conducted a detailed analysis at the QAP level (n=778). While the criteria “sleep disorder” and “depressive mood” were consistently well recognized, errors mainly occurred when students did not explore key aspects of the medical history in sufficient depth. This was particularly evident in the diagnostic criteria “feelings of guilt” and “reduced self-esteem.” It is noteworthy that misinterpretations of the AI-generated answers were rare (less than 2%) and mainly related to suicidal ideation. This suggests that diagnostic difficulties were due more to insufficient follow-up questions than to a misunderstanding of the AI answers.

### Neglecting Suicidal Tendencies

Even more concerning is that the majority of students (14/23, 60.87%) either did not address the topic of potential suicidality at all or did so only insufficiently—despite explicit instruction during the introduction. Our qualitative analysis shows that the LLM sometimes responded very avoidantly to questions about suicidality, which may have given students a false impression of the actual severity of the topic. However, the model never denied existing suicidality but was simply reluctant to talk about it, which adequately mirrors some real patients’ behavior. Future prompts should, nonetheless, contain clearer risk indicators to guide students toward more targeted questions and avoid making the scenarios too challenging, especially at such early career training stages. Additionally, although students knew that asking explicit questions about suicidal thoughts is a standard procedure in mental health assessment, many still found it difficult to address, causing considerable discomfort and uncertainty. Literature shows that suicidality is often a shameful topic for medical students, and they fear that addressing it will intensify suicidal thoughts [58,59]. However, the opposite is the case, which underscores the need for targeted training in how to deal with suicidality [58,60,61]. The integration of AI-supported virtual patients into mental health curricula, in combination with structured feedback sessions, could offer sustainable opportunities to practice assessing suicidality and reduce discomfort and uncertainty among students.

### Implications for AI-Supported Medical Training

Our results show that AI-supported online training for the diagnosis of depression and suicidality is a feasible and effective method. Nevertheless, we recommend integrating it into face-to-face formats—either fully, as in this study, or in a blended learning approach.

While other studies have shown that online training for suicide prevention can be equally effective or even superior [62,63], our results suggest that the immersive nature of virtual patient encounters contributes to a high level of perceived realism. In the feedback sessions, some students reported feeling emotionally affected, particularly when dealing with suicidality, which underscores the importance of structured support in such training formats.

The extent to which these observations can be generalized beyond our specific context remains to be investigated. Nevertheless, the potential for broader application in various medical education contexts appears promising.

### Limitations

This study has several limitations. First, it was conducted at a single medical school, limiting generalizability. Second, students had limited time to interact with the AI-supported patient, reflecting real-world time constraints but possibly insufficient for a practice scenario. Since the chatbot provides more information in a limited time than a real patient typically would, the diagnostic skills practiced may not fully transfer to real-world settings, and it must be assumed that performance in actual clinical encounters could be somewhat lower. Furthermore, the participants’ prior experience with AI-based systems was not assessed, which may have influenced their assessment of the tool and their interaction with it. This variable should be systematically evaluated in future studies.

Additionally, only 23 of 135 chats were analyzed at the QAP level. This selection was made because we aimed to maximize issue detection by focusing on chats in which students incorrectly assessed depression severity or suicidal ideation. This procedure ensured that the analyzed subset was both representative and information-rich. Nevertheless, analyzing full chat transcripts of incorrect diagnoses might have provided further insights.

Despite these limitations, this study provides valuable insights into the use of AI-based virtual patients to assess depression and suicidality. Future research should further develop chatbot models to support diagnostic training more comprehensively—in particular, through more precise and reliable feedback, improved conversational techniques (eg, question formulation and depth of exploration), and targeted support for clinical reasoning processes to better understand underlying thought patterns. Furthermore, it would also be interesting to examine the students’ reflections on the ethical aspects of interacting with chatbot patients.

### Conclusion

In summary, the chatbot can successfully simulate patients with depression with various stages of suicidality with high authenticity and adherence to predefined role scripts. Students’ diagnostic accuracy was influenced by the actual severity of depression, with moderate cases posing the greatest challenge. Notably, the majority either failed to address or insufficiently explored the topic of suicidality, which negatively influenced the correct classification of the phase of suicidality. AI-supported virtual patients provide a feasible and valuable tool for psychiatric history-taking training when cases are carefully developed and reviewed by experts. Potential risks, such as misinformation due to AI limitations and ethical considerations, should be addressed in future implementations.

Future medical education cannot be envisioned without integration of AI to practice realistic patient encounters—rigorous research on how these models should be programmed and used best is thus necessary. The AI tool presented here could benefit not only students and trainees but also general practitioners by increasing awareness of depression. Due to its online nature, it can be transferred to a variety of different settings and languages; nevertheless, it may require adaptation for less commonly used languages or specific cultural contexts. Ideally, this could contribute to improving the care of patients with depressive symptoms and thus to better management of this increasing societal challenge.

## Appendix

Multimedia Appendix 1 Additional material and prompts.

## Abbreviations

AI: artificial intelligence
DSM-5: Diagnostic and Statistical Manual of Mental Disorders, Fifth Edition
GPT: generative pretrained transformer
ICD-10: International Statistical Classification of Diseases, Tenth Revision
ICD-11: International Classification of Diseases, 11th Revision
LLM: large language model
PSM: psychosomatic medicine and psychotherapy
QAP: question-answer pair

## Data Availability

The datasets generated or analyzed during this study are available from the corresponding author (LH) on reasonable request.

## References

[1] GBD 2019 Diseases and Injuries Collaborators (2020). Global burden of 369 diseases and injuries in 204 countries and territories, 1990-2019: a systematic analysis for the Global Burden of Disease Study 2019. Lancet.

[2] Institute for Health Metrics and Evaluation (2023). GBD Results Tool.

[3] (2022). WHO Guidelines on Mental Health at Work. First edition.

[4] (2013). Diagnostic and Statistical Manual of Mental Disorders. Fifth edition.

[5] (1993). The ICD-10 Classification of Mental and Behavioural Disorders: Clinical Descriptions and Diagnostic Guidelines.

[6] Lynch CJ, Gunning FM, Liston C (2020). Causes and consequences of diagnostic heterogeneity in depression: paths to discovering novel biological depression subtypes. Biol Psychiatry.

[7] Handy A, Mangal R, Stead TS, Coffee RL, Ganti L (2022). Prevalence and impact of diagnosed and undiagnosed depression in the United States. Cureus.

[8] Pelletier L, O'Donnell S, Dykxhoorn J, McRae L, Patten SB (2016). Under-diagnosis of mood disorders in Canada. Epidemiol Psychiatr Sci.

[9] (2022). Depression in adults: treatment and management (NICE guideline NG222). National Institute for Health and Care Excellence.

[10] American Psychological Association (2019). Clinical Practice Guideline for the Treatment of Depression Across Three Age Cohorts.

[11] Arbeitsgemeinschaft der Wissenschaftlichen Medizinischen Fachgesellschaften (2023). Nationale VersorgungsLeitlinie Unipolare Depression – Langfassung, Version 3.

[12] (2023). Depressive disorder (depression). World Health Organization.

[13] Mojtabai R (2013). Clinician-identified depression in community settings: concordance with structured-interview diagnoses. Psychother Psychosom.

[14] Cepoiu M, McCusker J, Cole MG, Sewitch M, Belzile E, Ciampi A (2008). Recognition of depression by non-psychiatric physicians: a systematic literature review and meta-analysis. J Gen Intern Med.

[15] Houston K, Haw C, Townsend E, Hawton K (2003). General practitioner contacts with patients before and after deliberate self harm. Br J Gen Pract.

[16] Richards JC, Ryan P, McCabe MP, Groom G, Hickie IB (2004). Barriers to the effective management of depression in general practice. Aust N Z J Psychiatry.

[17] Levkovich I, Elyoseph Z (2023). Identifying depression and its determinants upon initiating treatment: ChatGPT versus primary care physicians. Fam Med Community Health.

[18] Coppens E, Van Audenhove C, Gusmão R, Purebl G, Székely A (2018). Effectiveness of General Practitioner training to improve suicide awareness and knowledge and skills towards depressionEffectiveness of general practitioner training to improve suicide awareness and knowledge and skills towards depression. J Affect Disord.

[19] van Os TWDP, van den Brink RHS, Jenner JA, van der Meer K, Tiemens BG, Ormel J (2002). Effects on depression pharmacotherapy of a Dutch general practitioner training program. J Affect Disord.

[20] Henriksson S, Isacsson G (2006). Increased antidepressant use and fewer suicides in Jämtland county, Sweden, after a primary care educational programme on the treatment of depression. Acta Psychiatr Scand.

[21] (2012). Public Health Action for the Prevention of Suicide: A Framework.

[22] (2014). Preventing Suicide: A Global Imperative.

[23] Yamamoto A, Koda M, Ogawa H, Miyoshi T, Maeda Y, Otsuka F (2024). Enhancing medical interview skills through AI-simulated patient interactions: nonrandomized controlled trial. JMIR Med Educ.

[24] Cook DA (2025). Creating virtual patients using large language models: scalable, global, and low cost. Med Teach.

[25] Potter L, Jefferies C (2024). Enhancing communication and clinical reasoning in medical education: building virtual patients with generative AI. Future Healthc J.

[26] Holderried F, Stegemann-Philipps C, Herschbach L, Moldt JA, Nevins A, Griewatz J (2024). A generative pretrained transformer (GPT)-powered chatbot as a simulated patient to practice history taking: prospective, mixed methods study. JMIR Med Educ.

[27] Holderried F, Stegemann-Philipps C, Herrmann-Werner A, Festl-Wietek T, Holderried M, Eickhoff C (2024). A language model–powered simulated patient with automated feedback for history taking: prospective study. JMIR Med Educ.

[28] Atapattu T, Thilakaratne M, Do DN, Herath M, Falkner KE, Che W, Nabende J, Shutova E, Pilehvar MT (2025). Exploring the role of mental health conversational agents in training medical students and professionals: a systematic literature review. Findings of the Association for Computational Linguistics: ACL 2025.

[29] Cook DA, Erwin PJ, Triola MM (2010). Computerized virtual patients in health professions education: a systematic review and meta-analysis. Academic Medicine.

[30] Foster A, Chaudhary N, Kim T, Waller JL, Wong J, Borish M (2016). Using virtual patients to teach empathy. Simul Healthc.

[31] Chaby L, Benamara A, Pino M, Prigent E, Ravenet B, Martin JC (2022). Embodied virtual patients as a simulation-based framework for training clinician-patient communication skills: an overview of their use in psychiatric and geriatric care. Front Virtual Real.

[32] Campillos-Llanos L, Thomas C, Bilinski É, Neuraz A, Rosset S, Zweigenbaum P (2021). Lessons learned from the usability evaluation of a simulated patient dialogue system. J Med Syst.

[33] Sezgin E, Chekeni F, Lee J, Keim S (2023). Clinical accuracy of large language models and Google search responses to postpartum depression questions: cross-sectional study. J Med Internet Res.

[34] Rosenman G, Wolf L, Hendler T LLM questionnaire completion for automatic psychiatric assessment. arXiv.

[35] Dupuy L, de Sevin E, Cassoudesalle H, Ballot O, Dehail P, Aouizerate B, Cuny E, Micoulaud-Franchi JA, Philip P (2020). Guidelines for the design of a virtual patient for psychiatric interview training. J Multimodal User Interfaces.

[36] (2023). DeepL Translator.

[37] Althaus D, Hegerl U (2004). Ursachen, Diagnose und Therapie von Suizidalität [Causes, diagnosis and treatment of suicidality]. Nervenarzt.

[38] Müssigbrodt H, Michels R, Malchow CP, Dilling H, Munk-Jørgensen P, Bertelsen A (2000). Use of the ICD-10 classification in psychiatry: an international survey. Psychopathology.

[39] Seitz L (2024). Artificial empathy in healthcare chatbots: does it feel authentic?. Comput Hum Behav Artif Hum.

[40] Braun V, Clarke V (2008). Using thematic analysis in psychology. Qualitative Research in Psychology.

[41] Chen S, Wu M, Zhu KQ, Lan K, Zhang Z, Cui L LLM-empowered chatbots for psychiatrist and patient simulation: application and evaluation. arXiv.

[42] Wang R, Milani S, Chiu JC, Zhi J, Eack SM, Labrum T PATIENT-Ψ: using large language models to simulate patients for training mental health professionals. arXiv.

[43] Wang C, Li S, Lin N, Zhang X, Han Y, Wang X (2025). Application of large language models in medical training evaluation—using ChatGPT as a standardized patient: multimetric assessment. J Med Internet Res.

[44] (2025). Character design. SillyTavern.

[45] Salewski L, Alaniz S, Rio-Torto I, Schulz E, Akata Z In-context impersonation reveals large language models’ strengths and biases. arXiv.

[46] Gupta S, Shrivastava V, Deshpande A, Kalyan A, Clark P, Sabharwal A Bias runs deep: implicit reasoning biases in persona-assigned LLMs. arXiv.

[47] Kessels RPC (2003). Patients' memory for medical information. J R Soc Med.

[48] Kühne F, Ay DS, Otterbeck MJ, Weck F (2018). Standardized patients in clinical psychology and psychotherapy: a scoping review of barriers and facilitators for implementation. Acad Psychiatry.

[49] Kühne F, Maaß U, Weck F (2021). Standardized patients in clinical psychology: from research to practice. Verhaltenstherapie.

[50] Barrows HS (1993). An overview of the uses of standardized patients for teaching and evaluating clinical skills. Acad Med.

[51] Kneebone R, Nestel D, Wetzel C, Black S, Jacklin R, Aggarwal R (2006). The human face of simulation: patient-focused simulation training. Acad Med.

[52] Lane C, Rollnick S (2007). The use of simulated patients and role-play in communication skills training: a review of the literature to August 2005. Patient Educ Couns.

[53] Krupinski J, Tiller JWG (2001). The identification and treatment of depression by general practitioners. Aust N Z J Psychiatry.

[54] Goldman LS, Nielsen NH, Champion HC (1999). Awareness, diagnosis, and treatment of depression. J Gen Intern Med.

[55] Kroenke K (2010). Review: GPs accurately diagnose about 50% of patients with depression and accurately classify 81% of nondepressed patients. Ann Intern Med.

[56] Gaebel W, Stricker J, Kerst A (2022). Changes from ICD-10 to ICD-11 and future directionsin psychiatric classification. Dialogues Clin Neurosci.

[57] Gaebel W, Stricker J, Riesbeck M, Zielasek J, Kerst A, Meisenzahl-Lechner E (2020). Accuracy of diagnostic classification and clinical utility assessment of ICD-11 compared to ICD-10 in 10 mental disorders: findings from a web-based field study. Eur Arch Psychiatry Clin Neurosci.

[58] Schulz P, Zapata I, Huzij T (2024). Examination of medical student and physician attitudes towards suicide reveals need for required training. Front Public Health.

[59] Jamison JM, Brady M, Fang A, Bùi Trà-My N, Wolk CB, Davis M (2025). A qualitative examination of clinician anxiety about suicide prevention and its impact on clinical practice. Community Ment Health J.

[60] Omerov P, Steineck G, Dyregrov K, Runeson B, Nyberg U (2014). The ethics of doing nothing: suicide-bereavement and research—ethical and methodological considerations. Psychol Med.

[61] Dazzi T, Gribble R, Wessely S, Fear NT (2014). Does asking about suicide and related behaviours induce suicidal ideation? What is the evidence?. Psychol Med.

[62] Holmes G, Clacy A, Hamilton A, Kõlves Kairi (2024). Online versus in-person gatekeeper suicide prevention training: comparison in a community sample. J Ment Health.

[63] Pechek AA, Vincenzes KA, Forziat-Pytel K, Nowakowski S, Romero-Lucero L (2024). Teaching suicide assessment and intervention online: a model of practice. Prof Couns.

[64] Suchikova Y, Tsybuliak N, Teixeira da Silva JA, Nazarovets S (2025). GAIDeT (Generative AI Delegation Taxonomy): a taxonomy for humans to delegate tasks to generative artificial intelligence in scientific research and publishing. Accountability Res.

